# High glucose augments angiotensinogen in human renal proximal tubular cells through hepatocyte nuclear factor-5

**DOI:** 10.1371/journal.pone.0185600

**Published:** 2017-10-20

**Authors:** Juan Wang, Yuki Shibayama, Hiroyuki Kobori, Ya Liu, Hideki Kobara, Tsutomu Masaki, Zhiyu Wang, Akira Nishiyama

**Affiliations:** 1 Department of Pharmacology, Faculty of Medicine, Kagawa University, Kagawa, Japan; 2 Department of Immuno-oncology, Fourth Hospital of Hebei Medical University, Shijiazhuang, P.R. China; 3 Departments of Pharmacology and of Nephrology, School of Medicine, International University of Health and Welfare, Tokyo, Japan; 4 Department of Gastroenterology and Neurology, Faculty of Medicine, Kagawa University, Kagawa, Japan; Tokushima University Graduate School, JAPAN

## Abstract

High glucose has been demonstrated to induce angiotensinogen (AGT) synthesis in the renal proximal tubular cells (RPTCs) of rats, which may further activate the intrarenal renin-angiotensin system (RAS) and contribute to diabetic nephropathy. This study aimed to investigate the effects of high glucose on AGT in the RPTCs of human origin and identify the glucose-responsive transcriptional factor(s) that bind(s) to the DNA sequences of AGT promoter in human RPTCs. Human kidney (HK)-2 cells were treated with normal glucose (5.5 mM) and high glucose (15.0 mM), respectively. Levels of AGT mRNA and AGT secretion of HK-2 cells were measured by real-time polymerase chain reaction (PCR) and enzyme-linked immunosorbent assay (ELISA), respectively. Consecutive 5’-end deletion mutant constructs and different site-directed mutagenesis products of human AGT promoter sequences were respectively transfected into HK-2 cells, followed by AGT promoter activity measurement through dual luciferase assay. High glucose significantly augmented the levels of AGT mRNA and AGT secretion of HK-2 cells, compared with normal glucose treatment. High glucose also significantly augmented AGT promoter activity in HK-2 cells transfected with the constructs of human AGT promoter sequences, compared with normal glucose treatment. Hepatocyte nuclear factor (HNF)-5 was found to be one of the glucose-responsive transcriptional factors of AGT in human RPTCs, since the mutation of its binding sites within AGT promoter sequences abolished the above effects of high glucose on AGT promoter activity as well as levels of AGT mRNA and its secretion. The present study has demonstrated, for the first time, that high glucose augments AGT in human RPTCs through HNF-5, which provides a potential therapeutic target for diabetic nephropathy.

## Introduction

Intrarenal renin-angiotensin system (RAS) plays a crucial role in development of diabetic nephropathy (DN) [1], which is the major renal complication of diabetes mellitus [2] as well as the main cause of end-stage renal disease [3]. Previous studies reported that intrarenal angiotensinogen (AGT) mRNA and angiotensin (Ang) II levels were enhanced in type 2 diabetic rats [4, 5]. This could further lead to renal proximal tubular hypertrophy and interstitial fibrosis by stimulating reactive oxygen species and eventually result in diabetic nephropathy [6, 7]. Furthermore, by using human kidney biopsied samples, Kamiyama *et al*. [8] have demonstrated that AGT mRNA and protein levels in human renal cortex proximal tubules were significantly augmented in patients with type 2 diabetes.

In the kidney, AGT is the only known substrate of renin as well as the rate-limiting factor for RAS activity, which is mainly expressed in renal proximal tubular cells (RPTCs) [9]. Kobori *et al*. [10] have manifested that AGT synthesis in RPTCs regulates intrarenal RAS activity for its contribution to renal accumulation of Ang II in rats. Liu *et al*. [11] have demonstrated that high glucose treatment induced much higher levels of apoptosis and caspase-3 activity in rat RPTCs overexpressing AGT in comparison to control RPTCs *in vitro*, and induction of diabetes in transgenic mice that overexpressing AGT in RPTCs led to significant increases in apoptosis of RPTCs compared with diabetic nontransgenic littermates, moreover, the above effects were markedly attenuated by insulin and/or RAS blockers. Therefore, the level of AGT in RPTCs plays an important role in the pathogenesis of DN.

In the case of rats, it has been reported that high glucose stimulated AGT synthesis in RPTCs and a glucose-responsive element located in the DNA sequences of AGT promoter has been identified [5]. As to human, Acres *et al*. [12] have succeeded in determining the region of human AGT promoter sequences. However, it is still unknown whether high glucose directly activates AGT synthesis in human RPTCs. In addition, the existence of glucose-responsive transcriptional factor(s) that bind(s) to AGT promoter sequences has not been identified.

Therefore, the present study aimed to investigate the effects of high glucose on AGT in the RPTCs of human origin and identify the glucose-responsive transcriptional factor(s) that bind(s) to the DNA sequences of AGT promoter in human RPTCs.

## Materials and methods

### Cell culture and conditioned medium

Human kidney (HK)-2 cells, an immortalized human RPTC line, were kindly gifted by Dr. Masaomi Nangaku (Tokyo University, Tokyo, Japan). HK-2 cells were grown in Dulbecco's modified eagle medium (DMEM; Life Technologies, Carlsbad, USA, catalog#11885084) supplemented with 10% fetal bovine serum (Nichirei Biosciences, Tokyo, Japan, catalog#17012) and 2% penicillin-streptomycin (5,000 U/mL; Gibco, Carlsbad, USA, catalog#15070063). Cells were maintained at 37°C in a humidified incubator with 5% CO_2_/95% air. Based on the experiment demands, HK-2 cells were treated with medium with different glucose concentrations (5.5, 8, 12, 15, 20 and 25 mM) as described previously [13, 14].

### Real-time PCR

HK-2 cells were lysed using ISOGEN (Nippon Gene, Toyama, Japan, catalog#319–90211) followed by RNA extraction with chloroform. RNA samples were reversely transcribed to cDNA using SuperScript III reverse transcriptase (Invitrogen, Carlsbad, USA, catalog#18080044). The cDNA served as the template for the following PCR reactions using specific human AGT primer sets (Table 1). Quantitative real-time PCR was performed using the real-time PCR system (Applied Biosystems, San Francisco, USA, catalog#7300), following manufacturers’ instructions. β-actin was used as an internal control.

**Table 1:** Nucleotide sequences of the primers used in this study.

**PCR** **(5’– 3’)**	**Forward primer**	**Reverse primer**
Human AGT	aactggtgctgcaaggatct	tctctctcatccgcttcaag
HNF-5*	gcgactctcaccaaggtctc	ggatgcaggcattgaaagat
**Mutagenesis** **(5’– 3’)**	**Forward primer**	**Reverse primer**
HNF-5*	catcctgaaggcattttggtgtctttcaatctggct	agccagattgaaagacaccaaaatgccttcaggatg
MEF2*	cccacccctcagcggcatcgtgaccc	gggtcacgatgccgctgaggggtggg
CREB*	ctgcctcacccactggatcactggcttctg	cagaagccagtgatccagtgggtgaggcag
**DNA sequencing** **(5’– 3’)**	**Primer sequence**	
HNF-5*	gcgactctcaccaaggtctc	
MEF2*	gagcagctgaaggtcacaca	
CREB*	catctcctggcctcaaaaag	

Asterisk (*) indicates binding sites of the transcriptional factor within human AGT promoter sequences.

### ELISA

Secreted AGT in the culture medium of HK-2 cells was measured using the human angiotensinogen assay kit (Immuno-Biological Laboratories, Gunma, Japan, catalog#27412) according to the protocol provided by the manufacturer as well as the previous description [15]. AGT levels were normalized by total cellular protein in the dish.

### Plasmid constructs

Seven consecutive 5’-end deletion mutant plasmid constructs of the region from −4358 to +122 (relative to the transcription start site of human AGT gene) around human AGT promoter sequences (AGT_−4,358/+122), which has been proved sufficient for the promoter activity in human RPTCs [12], were used as described in a previous study [12]. Mutations in binding sites of possible candidates of glucose-responsive transcriptional factor(s) were generated from intact human AGT_−4,358/+122 with the specific primer sets (Table 1) and the QuickChange site-directed mutagenesis kit (Agilent Technologies, California, USA, catalog#200519–5) according to manufacturer’s instructions. Whole sequences of all plasmid constructs were analyzed using the BigDye Terminator v3.1 cycle sequencing kit (Applied Biosystems, catalog#4336917), BigDye Terminator purification kit (Applied Biosystems, catalog#4376484) and genetic analyzer (Applied Biosystems, catalog#3130x1) based on the manuals provided by the manufacturers. Accuracy of the DNA sequences was confirmed as expected. The specific primers used for sequencing are listed in Table 1.

### Plasmid transfection

HK-2 cells were initially seeded into 24-well plates. After cell confluence reached to about 60%, cells were serum-starved for 24 h before transfection. For plasmid transfection, cells in each well were transfected with 250 ng of plasmid construct and 20 ng pRL-TK renilla luciferase reporter vector plasmid (Promega, Wisconsin, USA, catalog#E2241), as an internal control for transfection efficiency, using the Lipofectamine LTX reagent (Thermo Fisher Scientific, Carlsbad, USA, catalog#15338030) according to the protocol provided by the manufacturer.

### Dual luciferase assay

To measure human AGT promoter activity, dual luciferase assay was performed as described previously [16] using the Dual-luciferase reporter assay system kit (Promega, catalog#E1910) and microplate reader (Corona Electric, Wisconsin, USA, catalog#SH-9000), following the manuals from manufacturers. Data were normalized based on the value shown by the pRL-TK renilla luciferase activity.

### Bioinformatics

DNA sequences of AGT promoter located on human chromosome 1 were obtained from the National Center for Biotechnology Information (accession#NG008836.1). The motif search by using GENETYX software (Informer Technologies, Roseau, Dominica) allowed us to screen all transcriptional factors that corresponding to the glucose-responsive region (-22 to -1,896) of human AGT promoter sequences. According to the GENETYX software, transcriptional factors with scores ≧ 20, indicating possible binding to the glucose-responsive region, were determined. The likelihood of binding increases as the score increases. Transcriptional factors with scores ≧ 50 have the highest likelihood of binding to the glucose -responsive region.

### Chromatin immunoprecipitation

To measure the binding level of a transcriptional factor to human AGT promoter sequences, chromatin immunoprecipitation was performed, as described previously [17], using the EZ-CHIP chromatin immunoprecipitation kit (Merck Millipore, Darmstadt, Germany, catalog#17–371), and either HNF-5 (also called HNF-3 [18, 19]) monoclonal antibody (Santa Cruz, California, USA, catalog#sc-377033) or mouse IgG (Santa Cruz, catalog#sc-516176) as the negative control, according to the manufacturers’ instructions. Precipitated DNA was analyzed by PCR using the specific primer sets listed in Table 1.

### Statistical analysis

Data are presented as mean ± SEM. Unpaired *t* test was used to compare values between two groups and one-way ANOVA followed by the Tukey’s test was used to compare values among more than two groups. *P*-value <0.05 was considered statistically significant. All statistical analyses were performed using Prism 5 software (GraphPad, California, USA).

## Results

### High glucose augmented levels of AGT mRNA and AGT secretion of HK-2 cells

**Fig 1:**
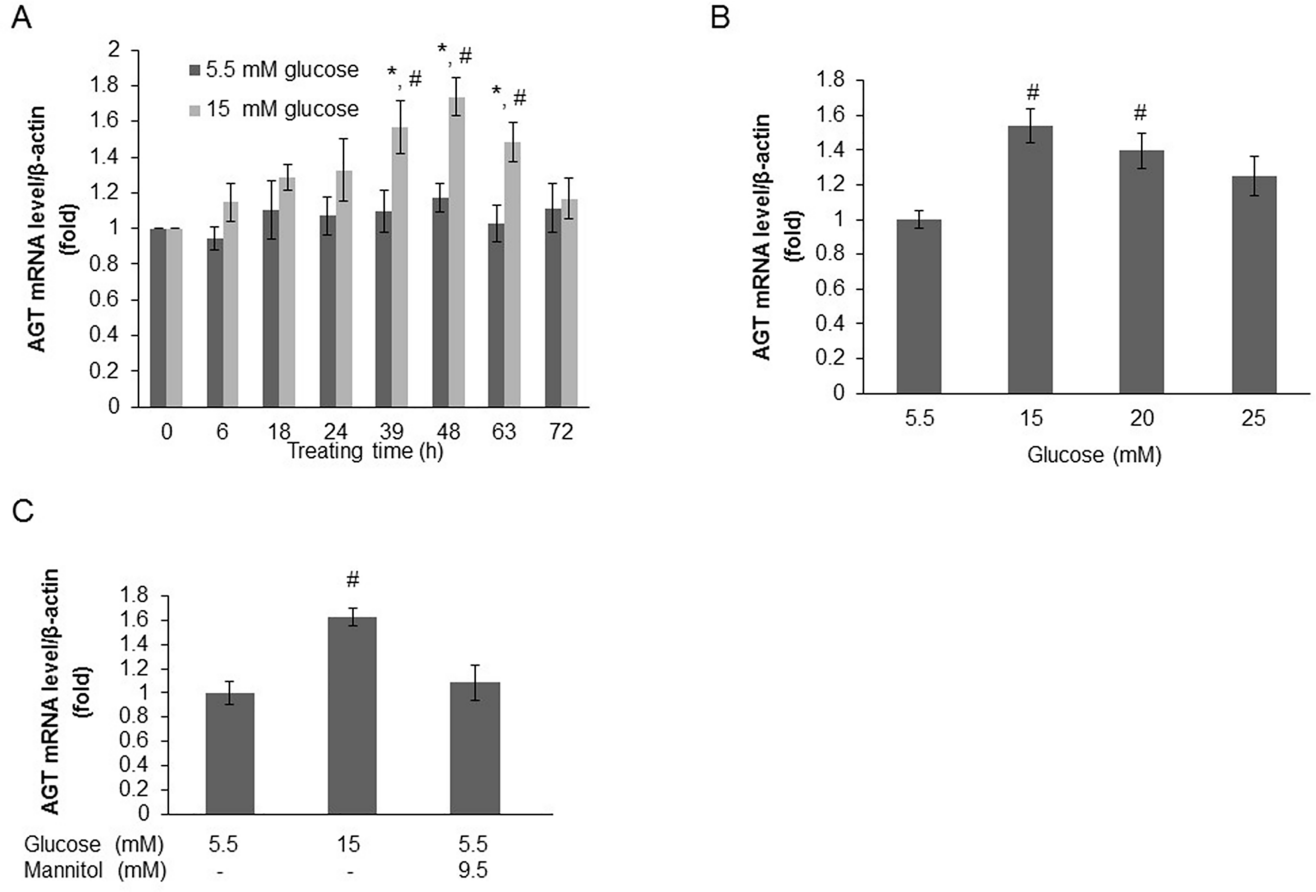
High glucose augments AGT mRNA level in HK-2 cells in a time- and dose-dependent manner. (A) AGT mRNA levels measured at different time points in HK-2 cells respectively treated with normal glucose (5.5 mM) and high glucose (15.0 mM). Compared with normal glucose treatment, high glucose significantly augmented AGT mRNA level from 39 h to 63 h. (B) AGT mRNA levels measured in HK-2 cells respectively treated with different glucose concentrations for 48 h. Compared with normal glucose treatment, high glucose augmented AGT mRNA level with the most significant effects by 15.0 or 20.0 mM glucose. (C) The effect of high glucose on AGT mRNA level was not due to osmotic stress which was further balanced with mannitol treatment for 48 h. Data are expressed as relative values to the 0h group (A) or normal glucose group (B and C). Values are presented as mean ± SEM. *P<0.05 *vs*. 0h group with the same glucose concentration; #*P*<0.05 *vs*. normal glucose group with the same treating time. N = 3~6.

To detect the time-dependent effects of high glucose on AGT mRNA levels, HK-2 cells were treated with either normal glucose (5.5 mM) or high glucose (15.0 mM), and AGT mRNA levels were measured at different time points. Compared with normal glucose treatment, high glucose significantly augmented AGT mRNA levels from 39 h (1.10±0.12 *vs*. 1.57±0.15, relative ratios to the 0 h group; *P*<0.05) to 63 h (1.03±0.10 *vs*. 1.48±0.11, relative ratios to the 0 h group; *P*<0.05). Treatment for 48 h (1.17±0.08 *vs*. 1.74±0.11, relative ratios to the 0 h group; *P*<0.01) was the most effective (Fig 1A). To detect the dose-dependent effects of high glucose on AGT mRNA levels, HK-2 cells were treated with different concentrations of glucose for 48 h. Compared with normal glucose treatment, 15.0 mM or 20.0 mM glucose significantly augmented AGT mRNA levels (15 mM: 1.00±0.05 *vs*. 1.54±0.10, relative ratios to the normal glucose group; *P*<0.001; 20 mM: 1.00±0.05 *vs*. 1.40±0.01, relative ratios to the normal glucose group; *P*<0.01). However, high glucose of 25.0 mM did not augment AGT mRNA level (1.00±0.05 *vs*. 1.25±0.11, relative ratios to the normal glucose group; *P* = 0.069) (Fig 1B). In the additional experiment, we also found that 8 mM, the marginal level of postprandial blood glucose in healthy subjects, is the threshold glucose concentration that can stimulate increased AGT (S1A Fig). To exclude the influence of osmotic stress, mannitol was used to equalize the total osmotic stress, which could be induced by both glucose and mannitol, in each group. As expected, mannitol treatment for 48 h did not influence AGT mRNA levels in HK-2 cells (Fig 1C). Therefore, the effect of high glucose on AGT mRNA level was not due to osmotic stress. Taken together, these data suggested that high glucose directly augmented AGT mRNA levels of HK-2 cells in a time- and dose-dependent manner.

Previous *in vivo* studies conducted in our lab have shown that the augmentation of intrarenal AGT was inhibited by treatment with an Ang II receptor blocker (ARB) in diabetes [4, 8]. Additional experiments were performed to examine the effect of an ARB (valsartan) on high glucose-induced augmentation of AGT in HK2 cells. We used valsartan at a concentration of 10 μM, as previously described [20, 21]. However, high glucose-induced augmentation of AGT was not affected by treatment with valsartan (S1B Fig).

**Fig 2:**
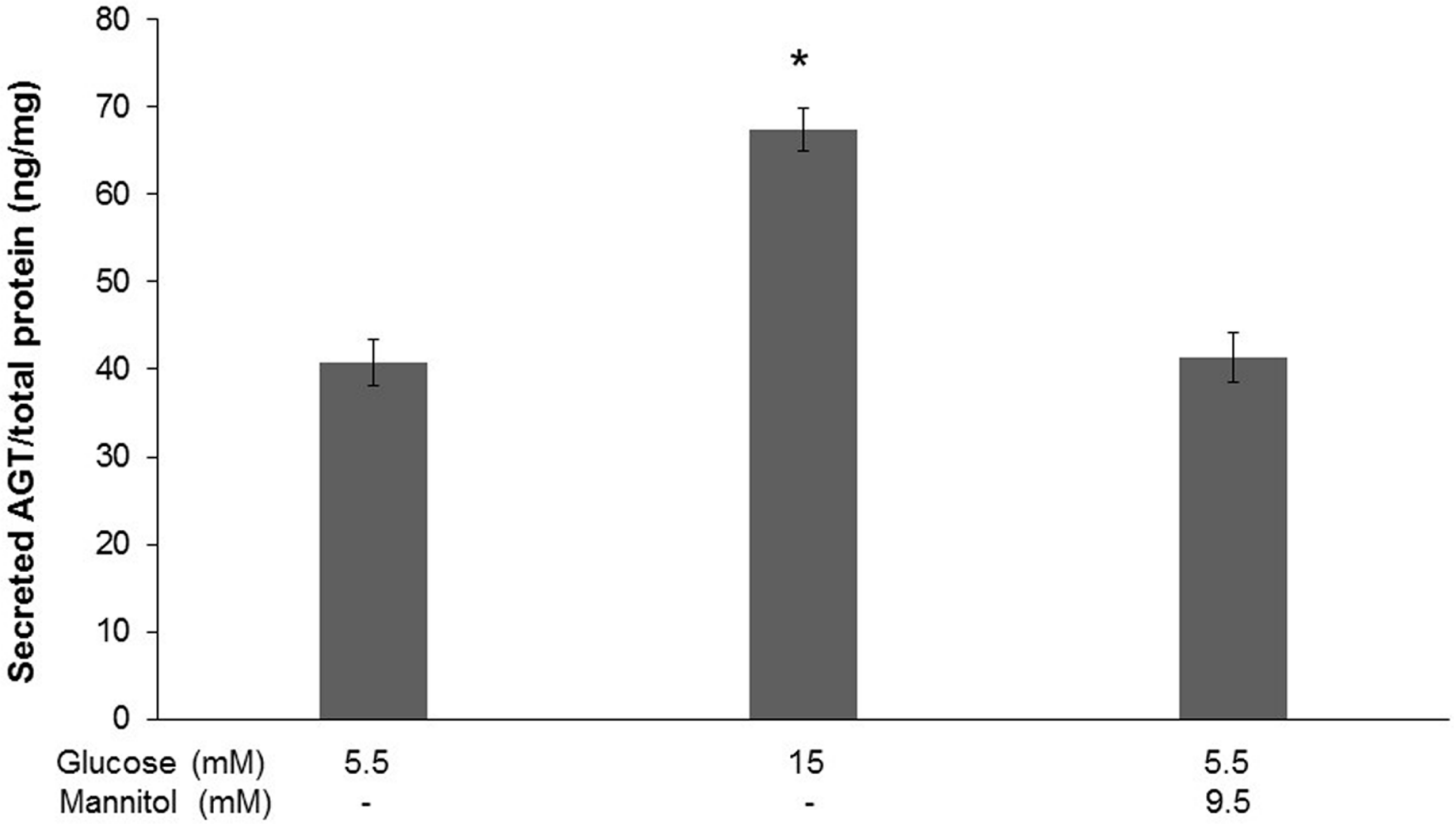
High glucose augments AGT secretion of HK-2 cells. Levels of secreted AGT in the culture medium of HK-2 cells respectively treated with normal glucose (5.5 mM), high glucose (15.0 mM) and normal glucose plus 9.5 mM mannitol. Compared with normal glucose treatment, high glucose significantly augmented AGT secretion into the medium, which was not affected by mannitol-induced osmotic stress. Data are expressed as relative values normalized by total cellular protein amount in the dish. Values are presented as mean ± SEM. *P<0.05 *vs*. normal glucose group. N = 3~6.

We further investigated the effects of high glucose on AGT secretion into the cell culture medium. Compared with normal glucose, high glucose (15.0 mM) treatment for 48 h significantly augmented secreted AGT levels (40.81±2.69 *vs*. 67.35±2.49 ng/mg total protein; *P*<0.0001), however, secretion of AGT was not affected by mannitol-induced osmotic stress (Fig 2).

### High glucose augmented AGT promoter activity through glucose-responsive region (-22 to -1,896) of human AGT promoter sequences

**Fig 3:**
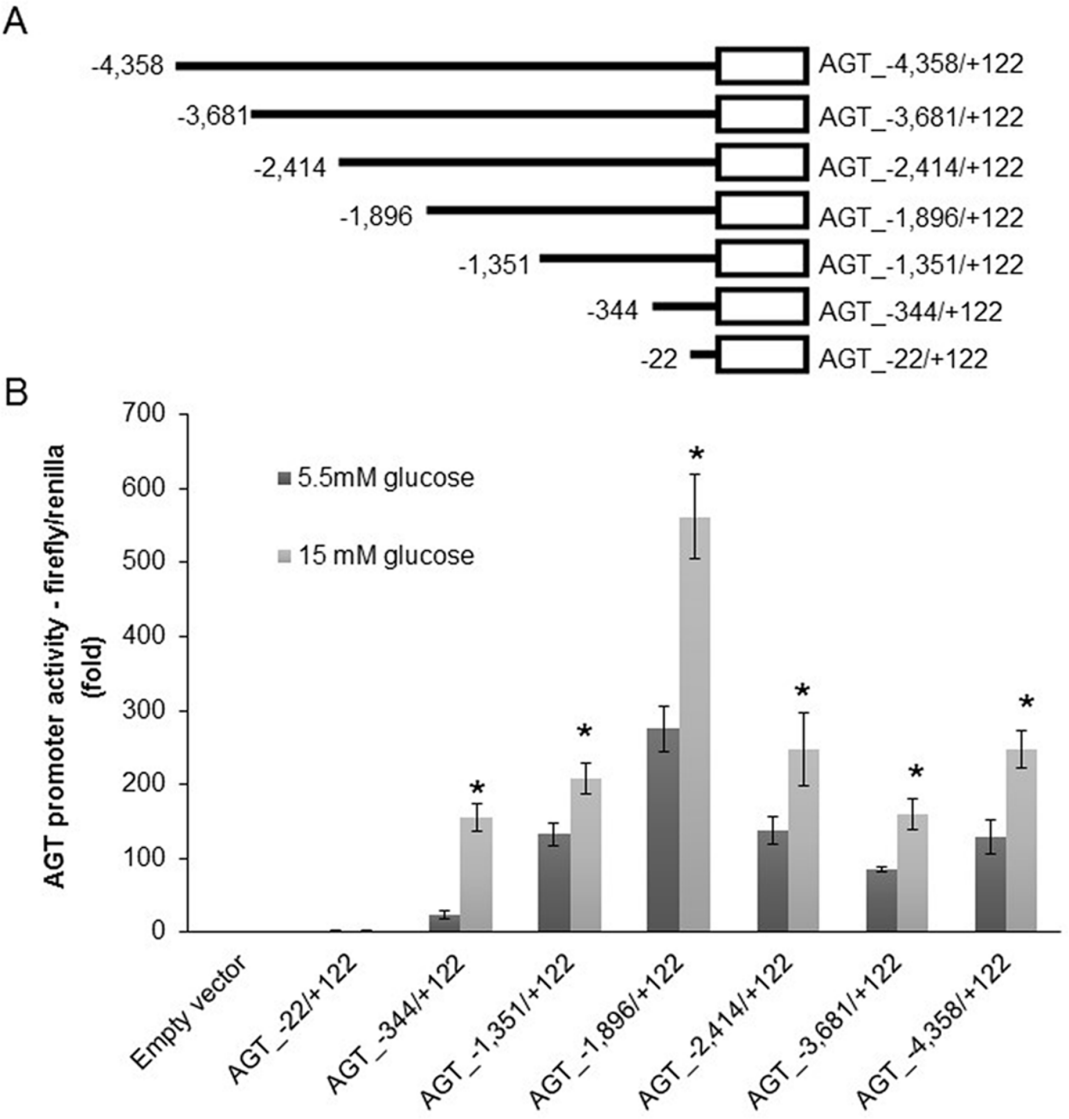
High glucose augments AGT promoter activity of HK-2 cells transfected with the promoter sequences constructs. (A) Consecutive 5’-end deletion mutant constructs of the DNA sequences of human AGT promoter used in this study. Horizontal line represents DNA sequences of the relevant deletion mutant construct. Open box represents the luciferase reporter. (B) Effects of high glucose on AGT promoter activity of HK-2 cells respectively transfected the constructs with 5’-ends from -22 to -4,358. Compared with normal glucose (5.5 mM) treatment, high glucose (15.0 mM) significantly augmented AGT promoter activity of HK-2 cells respectively transfected with the constructs with 5’-ends from -344 to -4,358. Additionally, within high glucose treatment groups, consecutive increase of AGT promoter activity was observed in HK-2 cells harboring constructs with 5’-ends from -344 to -1,896, while followed by marked decrease of AGT promoter activity in HK-2 cells harboring constructs with 5’-ends from -2,414 to -4,358. Data are expressed as relative values to the empty vector transfection group under the same glucose concentration. Values are presented as mean ± SEM. *P<0.05 *vs*. normal glucose group with the same construct. N = 3~6.

Seven consecutive 5’-end deletion mutant plasmid constructs of the DNA sequence region from −4358 to +122 of human AGT promoter (AGT_−4,358/+122), which has been proved sufficient for the promoter activity in human RPTCs [12], were employed (Fig 3A). Equal amounts of each construct as well as empty vector were respectively transfected into HK-2 cells, followed by the measurement of AGT promoter activity through dual luciferase assay. Compared with normal glucose (5.5 mM), high glucose (15.0 mM) treatment for 48 h significantly augmented AGT promoter activity of HK-2 cells transfected with the constructs with 5’-ends from -344 to -4,358 (hAGT_-344/+122: 23±5 *vs*. 155±18, *P*<0.0001; hAGT_-1,351/+122: 133±15 *vs*. 242±21, *P*<0.01; hAGT_-1,896/+122: 275±31 *vs*. 562±57, *P*<0.01; hAGT_-2,414/+122: 138±18 *vs*. 274±50, *P*<0.05; hAGT_-3,681/+122: 85±4 *vs*. 160±21, *P*<0.01; hAGT_-4,358/+122: 130±23 *vs*. 248±25, *P*<0.01; relative ratios to empty vector transfection group under the same glucose concentration) (Fig 3B). Additionally, within the high glucose treatment groups, consecutive increases of AGT promoter activity were observed in HK-2 cells harboring constructs with 5’-ends from -344 to -1,896, while followed by marked decreases of AGT promoter activity in HK-2 cells harboring constructs with 5’-ends from -2,414 to -4,358 (Fig 3B). These data suggested that binding sites of glucose-responsive transcriptional factor(s) may lie in the promoter region from -22 to -1,896, which was regarded as glucose-responsive region.

### Possible glucose-responsive transcriptional factor candidates

GENETYX software was used to screen all the corresponding transcriptional factors of the glucose-responsive region (-22 to -1,896) in human AGT promoter sequences. Among the transcriptional factors that might bind to the glucose-responsive region (score ≧20) (Tables 2–4), three ones were found to be both glucose-responsive and closely associated with human AGT regulation as described below. The hepatocyte nuclear factor (HNF) family including HNF-5 (site position: -634 to -639; score >50, Table 2) is extensively involved in AGT regulation and/or high glucose effects [22, 23]. A previous study reported that glucosamine can stimulate AGT mRNA expression by activating cAMP-responsive element-binding protein (CREB) (site position: -837 to -841; score>40, Table 3) in rat RPTCs [24]. Additionally, Feng *et al*. [25] reported that AGT mRNA expression was significantly higher in the hearts of diabetic rats compared with healthy rats, which is in association with increased myocyte enhancer factor (MEF)2 (site position: -32 to -41; score>20, Table 4). Based on above information, we determined HNF-5, CREB and MEF2 as glucose-responsive transcriptional factor candidates of the AGT promoter in human RPTCs.

**Table 2:** Corresponding transcriptional factors of human AGT promoter sequence region -344 to -1896 (score ≧ 50).

Score	Position	Motifs	Factors	Consensus sequence (5’–3’)	Sequence start site
50.89	-1818 to -1846	CAP-SITE	unknown	cacttt	-1833
		IE1.2	unknown	ctttcc	-1831
50.3	-1772 to -1815	NF-E1_CS1	GATA-1	attatctt	-1801
		GAL4-GAL1-1.6	GAL4	ttatc	-1800
		NF-E1.5	GATA-1	tatctt	-1799
		H2A_CONSERVED _US	unknown	tcattc	-1775
52.07	-1374 to -1406	C/EBP-TTRS3	C/EBP	tcttactc	-1382
		C/EBP_CS2	C/EBP	tcttactc	-1382
		GCN4-HIS3.4	GCN4	ttactc	-1380
		GAMMA-IRE_CS	unknown	ctgtagcc	-1375
53.25	-1130 to -1190	HINF-A_RS	HiNF-A	atttcagaattt	-1163
		BHLH_CS	multiple	catgtg	-1144
52.07	-621 to -654	HNF-5_CS	**HNF-5**	gtttgt	-634
		CAP-SITE	unknown	caatct	-621
58.58	-602 to -622	LBP-1_RS	LBP-1	tctgg	-618
		LBP-1_CS	LBP-1	tctgg	-618
51.48	-518 to -566	BHLH_CS	multiple	cacttg	-549
		SV40.11	unknown	cccag	-530

**Table 3:** Corresponding transcriptional factors of human AGT promoter sequence region -344 to -1896 (50 > score ≧ 40).

Score	Position	Motifs	Factors	Consensus sequence (5’–3’)	Sequence start site
42.01 ~ 50.89	-1772 ~ -1846	C-MYB_CS	c-Myb	cagttg	-1810
		BHLH_CS	multiple	cagttg	-1810
		NF-E1_CS1	GATA-1	attatctt	-1801
		GAL4-GAL1-1.6	GAL4	ttatc	-1800
		NF-E1.5	GATA-1	tatctt	-1799
49.11	-1672 ~ -1710	BHLH_CS	multiple	catatg	-1695
40.24 ~ 52.07	-1334 ~ -1444	C-MYC_RS1	c-Myc	tctctta	-1435
		C/EBP-TTRS3	C/EBP	tcttactc	-1382
		C/EBP_CS2	C/EBP	tcttactc	-1382
		GCN4-HIS3.4	GCN4	ttactc	-1380
43.79	-1274 ~ -1320	TCF-1_CS	TCF-1	aaaag	-1309
		E2A_CS	E2A	acagatg	-1294
		BHLH_CS	multiple	cagatg	-1293
		T-AG-SV40.2	T-Ag	gaggc	-1286
42.60 ~ 47.34	-1178 ~ -1256	TCF-1_CS	TCF-1	caaag	-1251, -1183
40.24 ~ 53.25	-1095 ~ -1175	MFA2.1	MAT-alpha-2	atgtattt	-1167
		HINF-A_RS	HiNF-A	atttcagaattt	-1163
		BHLH_CS	multiple	catgtg	-1144
		TCF-1_CS	TCF-1	aaaag	-1097
43.2	-1048 ~ -1075	AP1-IL2	AP-1	ttcagtcagt	-1068
		GCN4-HIS4.3	GCN4	cagtca	-1066
42.01 ~ 45.56	-706 ~ -1007	MCBF_RS	MCBF	cattcct	-994
		LF-A1_RS	LF-A1	tgaacc	-945
		LVC_RS	LVc	cctgc	-941, -853
		LVC-MO-MULV	LVc	cctgc	-941, -853
		MFA2.1	MAT-alpha-2	atgtattt	-887
		CRE.1	CREB	cgtca	-837
		BHLH_CS	multiple	cacttg/catgtg	-834, -716
		TCF-1_CS	TCF-1	aaaag	-767
41.42	-671 ~ -699	TCF-1_CS	TCF-1	cacag	-678
40.24 ~ 58.58	-566 ~ -634	LBP-1_RS	LBP-1	tctgg/actgg	-618, -575
		LBP-1_CS	LBP-1	tctgg/actgg	-618, -575
		TCF-1_CS	TCF-1	aacag	-593
		HSTF_CS2	HSTF	ctggaaacttccag	-574
		HSTF_CS1	HSTF	ctggaaacttccag	-574
		H-APF-1_RS	H-APF-1	ctggaaa	-574
		HSE_CS_INVERTED _REPEAT	HSTF	ctggaaacttccag	-574
		HSTF_CS3	HSTF	ggaaacttccagaag	-572

**Table 4:** Corresponding transcriptional factors of human AGT promoter sequence region -22 to -344 (score ≧ 20).

Score	Position	Motifs	Factors	Consensus sequence (5’–3’)	Sequence start site
21.89 to 35.50	-329 to -361	ER_HALF-SITE	ER	ggtca	-337
		SP1_CS4	Sp1	ggggctggg	-344
26.4	-254 to -299	LVC-MO-MULV	LVc	cctgc	-255
		LF-A1_RSLF-A1	LF-A1	tggccc	-266
		H-APF-1 _RSH-APF-1	H-APF-1	ctgggaa	-276
20.71	-204 to -234	LVC_RS	LVc	cctgc	-224
24.26	-124 to -168	LBP-1_CS/RS	LBP-1	tctgg	-124
		GR-MT-IIA GR	GR	tgtcct	-130
		BHLH_CS	multiple	catctg	-134
		TGGCA-BP_RS	TGGCA-BP	tggca	-137
26.63	-90 to -123	P7II_CS	P7II	gtaaccctc	-94
		IBP-1_CS2	IBP-1	aagtga	-101
		BHLH_CS	multiple	caagtg	-102
		LBP-1_RS	LBP-1	tctgg	-108
		LBP-1_CS	LBP-1	tctgg	-108
		CAP-SITE	unknown	cagcct	-118
24.26	-22 to -86	TATA-BOX-CS	TFIID	tataaat	-31
		TFIID/TBF-RS	TFIID/TBF	tataaa	-31
		B-FACTOR-HSP70	B-factor	tataaata	-31
		TFIID/TBP-H2B1	TFIID/TBP	tataaatag	-31
		HIS3-TR-TATA	TFIID	tataaa	-31
		TATA-BOX.2	TFIID	tataaa	-31
		MEF2_CS	**MEF2**	ctataaatag	-32
		CTCF_RS	CTCF	ccctc	-39
		AP-2_CS6	AP-2	ccccaccc	-45
		AP-2_CS4	AP-2	tccccacccc	-46

### Mutation in HNF-5 binding sites reduced the effects of high glucose on human AGT promoter activity as well as AGT mRNA and its secretion levels

**Fig 4:**
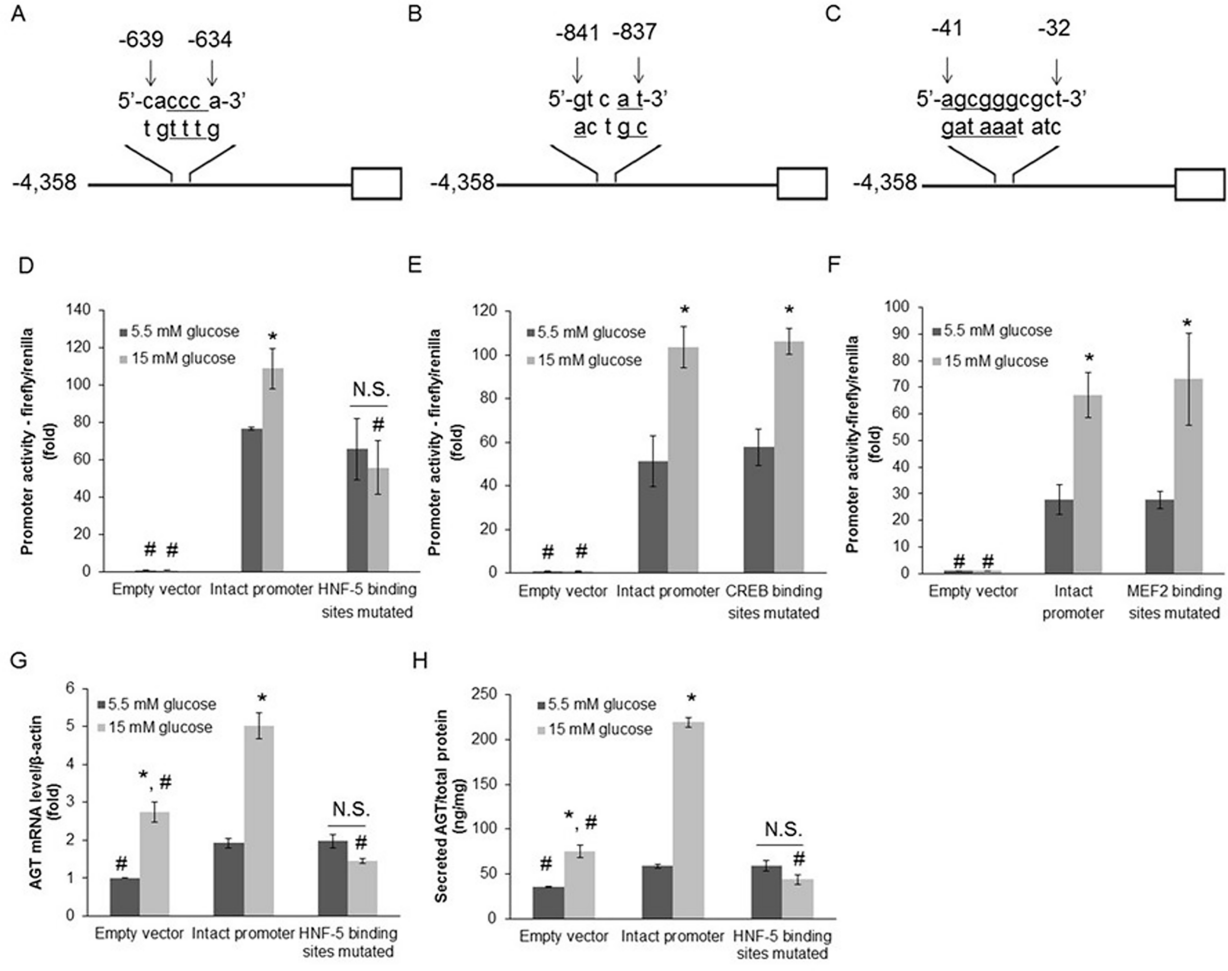
Mutation in HNF-5 binding sites reduces the effects of high glucose on AGT promoter activity as well as AGT mRNA and secretion levels. (A, B and C) Constructs with respective mutation of HNF-5 (A), CREB (B) or MEF2 (C) binding sites in main human AGT promoter sequences (AGT_-4,358/+122). Within binding sites, the underlined base pairs were substituted by mutagenesis. Horizontal line represents human AGT_-4,358/+122 with relevant mutation. Open box represents the luciferase reporter. (D, E and F) Effects of high glucose treatment on AGT promoter activity in HK-2 cells, which were transfected with either intact or relevant binding sites mutated construct of human AGT_-4,358/+122. Compared with normal glucose (5.5 mM) treatment, high glucose (15.0 mM) significantly augmented AGT promoter activity of HK-2 cells transfected with the intact construct of human AGT_-4,358/+122 (D, E and F). Furthermore, the above effect was abolished in HK-2 cells with mutated HNF-5 binding sites (D), while not affected by the binding sites mutation of CREB (E) or MEF2 (F). g: guanine; a: adenine; c: cytosine; t: thymine. Data are expressed as relative values to the empty vector transfection group under the same glucose concentration. (G) Mutation in HNF-5 binding sites attenuated the effects of high glucose on AGT mRNA level. Data are expressed as relative values to the empty vector transfection group with normal glucose. (H) Mutation in HNF-5 binding sites attenuated the effects of high glucose on AGT secretion level. Values are presented as mean ± SEM. *P<0.05 vs. normal glucose (5.5 mM) group with the same construct; #*P*<0.05 vs. intact human AGT_-4,358/+122 transfection group under the same glucose concentration; N.S.: no significant difference. N = 3~6.

Constructs with respective mutations of HNF-5 (Fig 4A), CREB (Fig 4B) or MEF2 (Fig 4C) binding sites in human AGT promoter sequences (AGT_-4,358/+122) were prepared. Equal amounts of empty vector, vector harboring either intact human AGT_-4,358/+122 or binding sites mutated human AGT_-4,358/+122 were respectively transfected into HK-2 cells, followed by AGT promoter activity measurement. Compared with normal glucose (5.5 mM), high glucose (15 mM) treatment for 48 h significantly augmented AGT promoter activity of HK-2 cells with intact human AGT_-4,358/+122 transfection (77±1 vs. 109±11, relative ratios to empty vector transfection group under the same glucose concentration; *P*<0.05) (Fig 4D). However, similar effect was not observed in HK-2 cells with mutated HNF-5 binding sites (66±17 vs. 56±14, relative ratios to empty vector transfection group under the same glucose concentration; *P* = 0.68) (Fig 4D). Different from the mutation of HNF-5 binding sites, mutations in the binding sites of CREB (58±8 vs. 106±6, relative ratios to empty vector transfection group under the same glucose concentration; *P*<0.01) and MEF-2 (28±3 vs. 73±17, relative ratios to empty vector transfection group under the same glucose concentration; *P*<0.05) had no effect on the response of AGT promoter activity to high glucose treatment (Fig 4E and 4F). Next, we investigated whether mutation in HNF-5 binding sites could attenuate AGT mRNA expression and its secretion under high glucose condition. In general, compared with groups transfected with the empty vector, those transfected with intact human AGT_-4,358/+122 showed much higher AGT mRNA and protein levels, especially under the high glucose condition (Fig 4G and 4H). These results suggest that the plasmid DNA could be integrated into the genome of HK-2 cells after transfection, moreover, the integrated promoter sequences even occupied a considerable proportion and were able to control AGT mRNA and protein expression together with the native AGT promoter sequences in HK-2 cells. Compared with normal glucose (5.5 mM), high glucose (15 mM) treatment for 48 h significantly augmented AGT mRNA levels of HK-2 cells transfected with either empty vector (1±0.01 vs. 2.74±0.27, relative ratios to the empty vector transfection group with normal glucose; *P*<0.001) or intact human AGT_-4,358/+122 transfection (1.93±0.13 vs. 5.02±0.35, relative ratios to the empty vector transfection group with normal glucose; *P*<0.001) (Fig 4G). However, this effect was not observed in HK-2 cells transfected with HNF-5 binding sites mutated AGT_-4,358/+122 (1.97±0.17 vs. 1.44±0.06, relative ratios to the empty vector transfection group with normal glucose; *P* = 0.10) (Fig 4G). Similar data were observed for AGT secretion levels. Compared with normal glucose (5.5 mM), high glucose (15 mM) treatment for 48 h significantly augmented AGT protein levels in the medium of HK-2 cells transfected with either empty vector (35.38±0.83 vs. 75.23±6.54 ng/mg total protein; *P*<0.0001) or intact human AGT_-4,358/+122 transfection (58.82±2.29 vs. 219.37±5.17 ng/mg total protein; *P*<0.0001) (Fig 4H). However, these effects were not observed in HK-2 cells transfected with HNF-5 binding sites mutated AGT_-4,358/+122 (59.12±5.49 vs. 43.72±5.65 ng/mg total protein; *P* = 0.12) (Fig 4H).

### Binding level of HNF-5 to AGT promoter sequences was reduced by mutation in its binding sites and elevated by high glucose treatment

**Fig 5:**
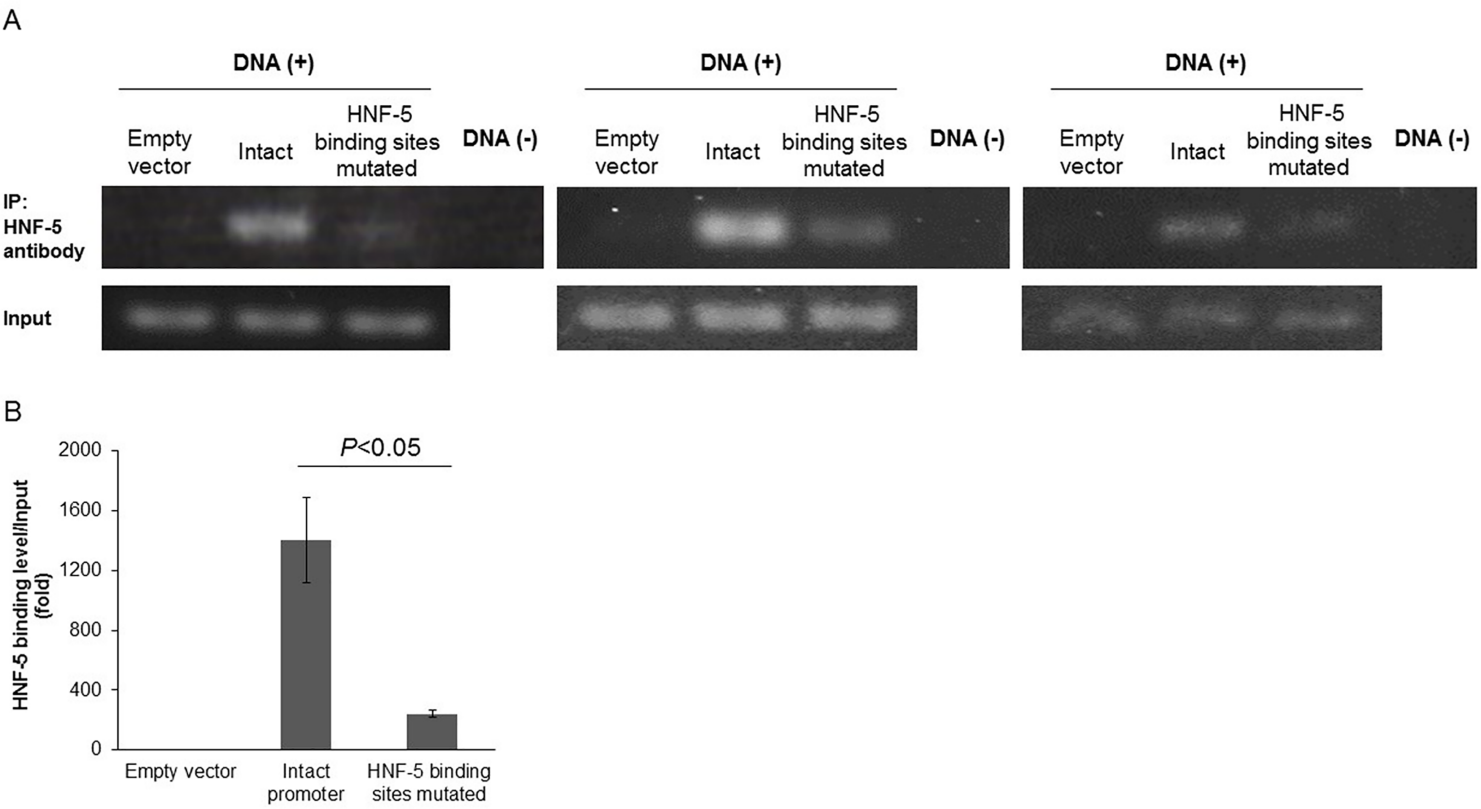
Binding level of HNF-5 to AGT promoter sequences is reduced by mutation in its binding sites. (A and B) Levels of DNA immuno-precipitated by HNF-5 protein from HK-2 cells respectively transfected with empty vector, intact and HNF-5 binding sites mutated main human AGT promoter sequences (AGT_-4,358/+122), detected through band intensities after PCR (A) and quantitative real-time PCR (B). Compared with the group with intact human AGT_-4,358/+122, the binding level of HNF-5 protein to human AGT_-4,358/+122 significantly decreased in HK-2 cells with HNF-5 binding sites mutation. Data of quantitative real-time PCR are expressed as relative values to the empty vector transfection group. DNA (−) indicates the absence of DNA, IP: immuno-precipitation. Values are presented as mean ± SEM. N = 3~6.

Equal amounts of empty vector, vector harboring intact human AGT_-4,358/+122 and vector harboring HNF-5 binding sites mutated human AGT_-4,358/+122 was transfected into HK-2 cells, respectively. Then the binding levels of HNF-5 protein to human AGT promoter sequences were detected by chromatin immunoprecipitation. As shown in at least 3 independent experiments, compared with the intact human AGT_-4,358/+122 transfection groups, HK-2 cells with HNF-5 binding sites mutation showed significantly attenuated HNF-5 binding levels. This was detected through both band intensities after PCR (Fig 5A) and quantitative real-time PCR (1402±287 vs. 240±23, relative ratios to the empty vector transfection group; *P*<0.05) (Fig 5B). These data confirm that mutation in HNF-5 binding sites reduced the binding level of HNF-5 to AGT promoter sequences. Additionally, we also measured the binding level of HNF-5 to the native genome of HK-2 cells without plasmid transfection. HNF-5 binding level was very low under normal glucose (5.5 mM), however, high glucose (15.0 mM) significantly augmented HNF-5 binding level. This was detected through both band intensities after PCR (S2A Fig) and quantitative real-time PCR (1±0.01 vs. 20.52±2.64, relative ratios to the normal glucose group; *P*<0.05) (S2B Fig).

## Discussion

Three novel findings were uncovered in the present study. First, high glucose augments the levels of AGT mRNA and AGT secretion of human RPTCs. Second, high glucose augments AGT promoter activity in human RPTCs and the glucose-responsive region within human AGT promoter sequences is from -22 to -1,896. Third, HNF-5 is one of the glucose-responsive transcriptional factors which bind to the glucose-responsive region of AGT promoter sequences and mutation of its binding sites may abolish the high glucose effects on AGT in human RPTCs

Intrarenal AGT is primarily generated in RPTCs and secreted into tubular fluid [10, 26]. Visavadiya *et al*. [27] have shown that high glucose can increase the expression levels of the RAS components including renin, angiotensin-converting enzyme as well as Ang II levels in HK-2 cells. In the present study, we aim to determine the detailed molecular mechanism responsible for high glucose-induced proximal tubular augmentation of AGT levels. We found that high glucose significantly augmented AGT mRNA levels in human RPTCs with the most obvious effects by 48 h- treatment with 15 or 20 mM glucose, which is similar to the blood glucose level of diabetic patients. Consistent findings were previously discovered in the case of rats, it is reported that high glucose stimulated AGT gene expression in rat RPTCs, which subsequently resulted in cell hypertrophy [13, 14]; On the other hand, stable transfection of antisense rat AGT cDNA into rat RPTCs effectively inhibited AGT expression and prevented high glucose-induced cell hypertrophy [28]. However, high glucose-induced augmentation of AGT in HK-2 cells was not affected by treatment with valsartan. We have no good explanation as to why ARB does not inhibit high glucose-induced augmentation of AGT *in vitro*, however, it is probably due to the low renin activity and AGT utilizing efficiency in HK-2 cells.

Additionally, we also found that high glucose significantly augmented AGT secretion from human RPTCs. AGT secreted from proximal tubules is regarded as the main source of urinary AGT [29]. Moreover, urinary AGT level reflects the activity of intrarenal RAS [30, 31] and tubular injury [32] in patients with type 2 diabetes. Elevation of urinary AGT has also been confirmed to be related to not only development but also progression of DN based on studies in patients with DN [31, 33], as well as mouse [34, 35] and rat [6, 36] models of diabetes. Therefore, finding in the present study suggests that high glucose-induced AGT secretion from RPTCs may play an important role in the pathogenesis of DN.

Previous studies have demonstrated that proximal promoter elements of human AGT genes from −4358 to +122 are sufficient to measure AGT promoter activity in human RPTCs [12, 37]. Furthermore, the importance of proximal regulatory elements to human AGT regulation has been confirmed by analyses with deletion mutants of AGT promoter sequences [12, 37]. Based on the previous study [12], we also employed the region from -4358 to +122 within human AGT promoter sequences and further identified its glucose-responsive region in human RPTCs. We found that high glucose treatment significantly augmented AGT promoter activity in HK-2 cells respectively transfected with the deletion mutant constructs with 5’-ends from -344 to -4,358. These data indicated that high glucose activates AGT by increasing AGT promoter activity in human RPTCs. Furthermore, within the high glucose treatment groups, consecutive increases of AGT promoter activity were observed in HK-2 cells harboring constructs with 5’-ends from -344 to -1,896, while followed by the marked decreases of AGT promoter activity in HK-2 cells harboring constructs with 5’-ends from -2,414 to -4,358. These data indicated that the glucose-responsive region, where glucose-responsive transcriptional factor(s) bind(s), should be from -22 to -1,896 of human AGT promoter sequences.

**Fig 6:**
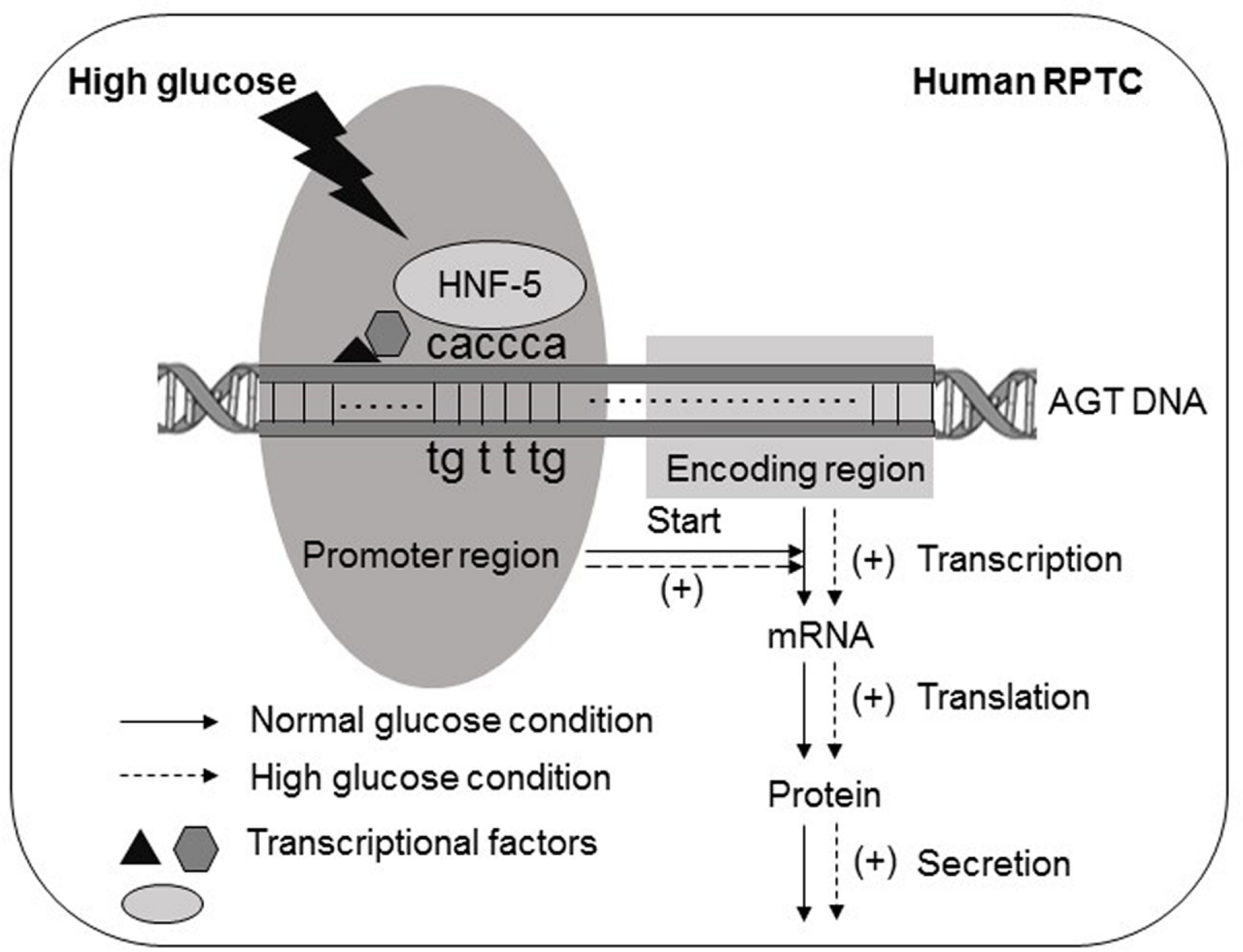
Schematic diagram summarizing the role of HNF-5 in AGT augmentation by high glucose in human RPTCs. In human RPTCs, through HNF-5, high glucose enhances the activity of AGT gene promoter, thus increases the levels of transcription, translation and secretion of AGT, in result, the levels of AGT mRNA and AGT secretion were augmented. g: guanine; a: adenine; c: cytosine; t: thymine.

Among all the transcriptional factors that possibly bind to the glucose-responsive region of human AGT promoter sequences (score ≧ 20), regulatory effect on AGT promoter activity in human RPTCs was observed in HNF-5. Soon after HNF-5 was initially designated [38], Nitsch *et al*. [18] demonstrated that HNF-5 is identical to HNF-3, because the HNF-3 binding sites within gene promoter sequences, identified by them, coincides with those of HNF-5. In the following literatures, both names were kept and applied to refer to the same factor [19]. HK-2 cells transfected with the HNF-5 binding sites mutated human AGT_-4,358/+122 did not show any increase in AGT promoter activity as well as AGT mRNA and its secretion levels under high glucose treatment. In other words, mutation of HNF-5 binding sites in human AGT promoter sequences abolished the high glucose effects on AGT in human RPTCs. Moreover, we also confirmed that binding level of HNF-5 to AGT promoter sequences was reduced by mutation in its binding sites and elevated by high glucose treatment. All of the above results uncovered the molecular mechanism that in human RPTCs, through HNF-5, high glucose enhances the activity of the AGT gene promoter, thus increasing the levels of transcription and secretion of AGT. As a result, the levels of AGT mRNA and AGT secretion were augmented (Fig 6). It has been well known that the HNF family is extensively involved in human AGT regulation, for example, HNF-1α [39] and HNF-4 [23] can bind to the AGT promoter sequences and activate AGT expression in human hepatoma cells. In the present study, we demonstrated, for the first time, that HNF-5 is one of the glucose-responsive transcriptional factors that bind to the AGT promoter sequences in human RPTCs. As far as we know, HNF-5 also plays a role as a glucocorticoid-responsive transcriptional factor in hepatoma cells [40]. However, in hepatoma cells, glucose-induced AGT augmentation is mediated predominantly through HNF-1α- and HNF-4-dependent pathways [23, 39]. Further studies are needed to determine the possible role of HNF-5 in AGT production in other organs.

As shown in Fig 4E and 4F, mutation of CREB or MEF2 binding sites within AGT promoter sequences did not abolish the high glucose-induced enhancement of AGT promoter activity in HK-2 cells. These data suggest that CREB and MEF2 does not contribute to augmenting AGT expression in RPTCs under high glucose condition. Using GENETYX software and published data, candidates for glucose-responsive transcriptional factors were selected based on their likelihood of binding to human AGT promoter sequences. There may be other glucose-responsive transcriptional factors that work on AGT promoter, but we omitted them due to the lack of published supporting evidence for their potential involvement in high glucose-induced effects and/or AGT regulation. HNF-5 is located from -634 to -639. However, as shown in Fig 3B, AGT promoter activity apparently stepped-up from AGT_-1,351/+122 to AGT -1,896/+122, suggesting that the sequences between -1,351 and -1,896 may play an important role in amplifying AGT induction under high glucose condition. Therefore, other glucose-responsive transcriptional factors that bind to this region should be investigated further.

In conclusion, the present study has demonstrated, for the first time, that high glucose augments AGT in human RPTCs through HNF-5, a glucose-responsive transcriptional factor that functions on the AGT gene promoter, which provides a potential therapeutic target for DN in humans.

## Supporting information

S1 Fig Augmenting effect of high glucose on AGT mRNA in HK-2 cells, with the threshold effective concentration of 8 mM, could not be affected by valsartan. (A) AGT mRNA levels measured in HK-2 cells respectively treated with different glucose concentrations for 48 h. Compared with normal glucose (5.5 mM) treatment, high glucose augments AGT mRNA level, with the threshold effective concentration of 8 mM. (B) The effect of high glucose on AGT mRNA level could not be affected by valsartan. There is no difference between AGT mRNA levels in HK-2 cells respectively treated with either high glucose (15 mM) or high glucose plus 10 μM valsartan for 48 h. Data are expressed as relative values to the corresponding normal glucose group. Values are presented as mean ± SEM. #*P*<0.05, ##*P*<0.01, ###*P*<0.001 vs. normal glucose group. N.S.: no significant difference. N = 3~6.
(TIF)

S2 Fig Binding level of HNF-5 to AGT promoter sequences in genome of HK-2 cells is elevated by high glucose treatment. (A and B) Levels of DNA immuno-precipitated by HNF-5 protein from native genome of HK-2 cells respectively treated with normal (5.5 mM) and high (15 mM) glucose, detected through band intensities after PCR (A) and quantitative real-time PCR (B). HK-2 cells were without plasmid transfection. Compared with normal glucose treatment, high glucose significantly augmented HNF-5 binding levels. Data of quantitative real-time PCR are expressed as relative values to the normal glucose group. DNA (−) indicates the absence of DNA, IP: immuno-precipitation, NG: normal glucose; HG: high glucose. Values are presented as mean ± SEM. N = 3~6.
(TIF)
