# Supplementary figures and images for: High glucose augments angiotensinogen in human renal proximal tubular cells through hepatocyte nuclear factor-5
Source: PLoS One. 2017 Oct 20;12(10):e0185600. doi: 10.1371/journal.pone.0185600 (PMC5650141; doi:10.1371/journal.pone.0185600)

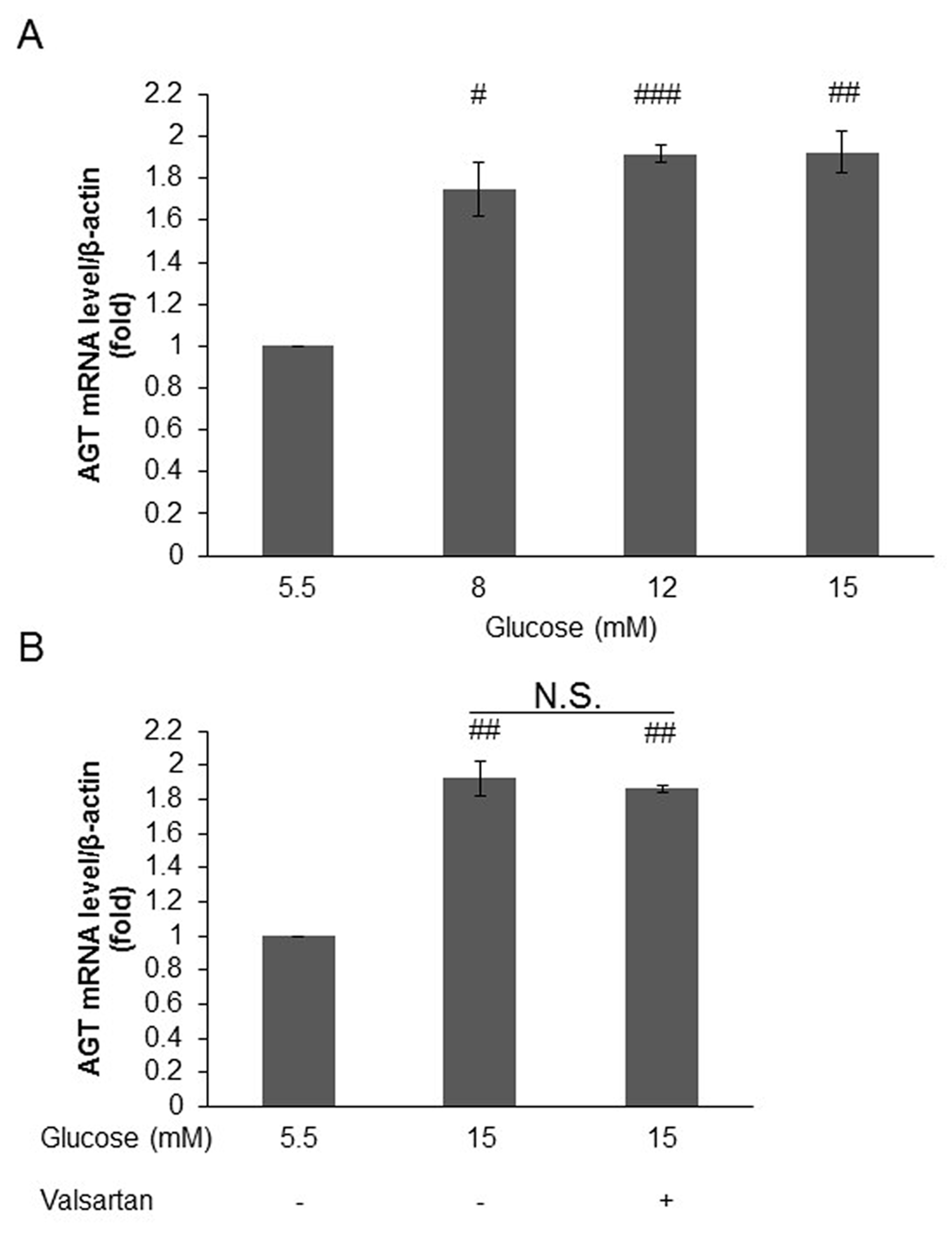

Supplement: S1 Fig — (A) AGT mRNA levels measured in HK-2 cells respectively treated with different glucose concentrations for 48 h. Compared with normal glucose (5.5 mM) treatment, high glucose augments AGT mRNA level, with the threshold effective concentration of 8 mM. (B) The effect of high glucose on AGT mRNA level could not be affected by valsartan. There is no difference between AGT mRNA levels in HK-2 cells respectively treated with either high glucose (15 mM) or high glucose plus 10 μM valsartan for 48 h. Data are expressed as relative values to the corresponding normal glucose group. Values are presented as mean ± SEM. #P<0.05, ##P<0.01, ###P<0.001 vs. normal glucose group. N.S.: no significant difference. N = 3~6. (TIF) [file pone.0185600.s001.tif]

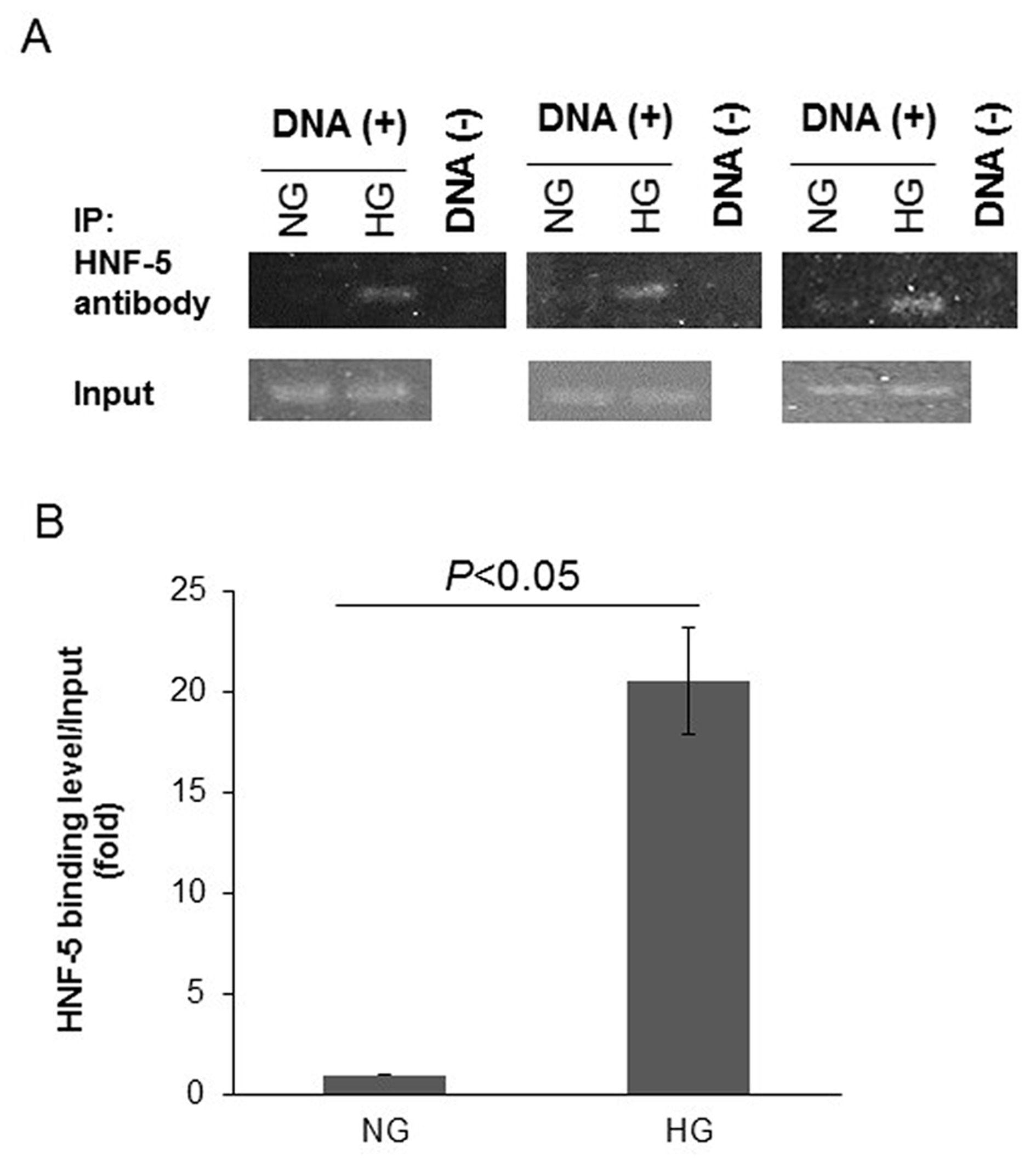

Supplement: S2 Fig — (A and B) Levels of DNA immuno-precipitated by HNF-5 protein from native genome of HK-2 cells respectively treated with normal (5.5 mM) and high (15 mM) glucose, detected through band intensities after PCR (A) and quantitative real-time PCR (B). HK-2 cells were without plasmid transfection. Compared with normal glucose treatment, high glucose significantly augmented HNF-5 binding levels. Data of quantitative real-time PCR are expressed as relative values to the normal glucose group. DNA (−) indicates the absence of DNA, IP: immuno-precipitation, NG: normal glucose; HG: high glucose. Values are presented as mean ± SEM. N = 3~6. (TIF) [file pone.0185600.s002.tif]
